# Overexpression of BDNF in the ventrolateral periaqueductal gray regulates the behavior of epilepsy–migraine comorbid rats

**DOI:** 10.1002/brb3.2594

**Published:** 2022-05-12

**Authors:** Long Wang, Lu‐Lan Fu, Zi‐Ru Deng, Juan Zhang, Mei‐Dan Zu, Jun‐Cang Wu, Yu Wang

**Affiliations:** ^1^ Department of Neurology The Second People's Hospital of Hefei Hefei China; ^2^ Department of Neurology The First Affiliated Hospital of Anhui Medical University Hefei China

**Keywords:** BDNF, comorbidity, epilepsy, migraine, ventrolateral periaqueductal gray

## Abstract

**Objective:**

To investigate the effects of brain‐derived neurotrophic factor (BDNF) overexpression in the ventrolateral periaqueductal gray (vlPAG) on behavioral changes in epilepsy–migraine comorbid rats.

**Method:**

We used an adeno‐associated virus (AAV)‐mediated vector to supplement BDNF in the vlPAG area prior to the establishment of a pilocarpine‐nitroglycerin (Pilo‐NTG) combination‐induced comorbid model of epilepsy and migraine. Seizure‐ and migraine‐related behaviors were analyzed. Cell loss and apoptosis in vlPAG were detected through hematoxylin‐eosin (HE) and TUNEL staining. Immunofluorescence staining analyses were employed to detect expressions of BDNF and its receptor, tyrosine kinase B (TrkB), in vlPAG. Immunohistochemical staining was conducted to detect expressions of c‐Fos and calcitonin gene‐related peptide (CGRP) in the trigeminal nucleus caudalis (TNC) and trigeminal ganglion (TG).

**Results:**

Comparing to control group, AAV–BDNF injected comorbid group showed lower pain sensitivity, scratching head, and spontaneous seizures accompanied by the downregulation of c‐Fos labeling neurons and CGRP immunoreactivity in the TNC and TG. However, these changes were still significantly higher in the comorbid group than those in both epilepsy and migraine groups under the same intervention.

**Conclusion:**

These data demonstrated that supplying BDNF to vlPAG may protect structural and functional abnormalities in vlPAG and provide an antiepileptic and analgesic therapy.

## Graphical abstract

The important finding is that BDNF overexpression in the vlPAG region highly significantly decreases the frequency and duration of spontaneous seizures as well as pain sensitivity in the epilepsy–migraine comorbid rat. This suggested that protective intervention for vlPAG may help to reduce the risk of nociceptive behavior caused by epileptic seizures, and we believe the underlying mechanism is significant.

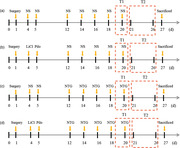

## 1 INTRODUCTION

Migraine is a common comorbidity of the nervous system in epilepsy (De Simone et al., 2007), and both have overlapping pathogenesis including neuroelectrophysiological disorders in the cerebral cortex and brainstem (Kim & Lee, 2017; Porcaro et al., 2017).

Periaqueductal gray (PAG) is a core region that is involved in the regulation of both endogenous pain and trigeminovascular pathway of pain (La Cesa et al., 2014; Vecchia & Pietrobon, 2012). It was found that PAG lesions, especially the structural lesions of vlPAG, can lead to excessive excitability of trigeminal vascular neurons, thus triggering repeated migraine attacks (Ito et al., 2016; Wang & Wang, 2013). Evidences from animal studies suggested that vlPAG plays a critical analgesic effect in postictal antinociception (de Freitas et al., 2014; Jaseja, 2013), as well as the pain sensitivity decreases during the interphase among people with chronic epilepsy (Szucs et al., 2015). Our recent study showed that the frequency and duration of epileptic seizures as well as pain sensitivity in rats with neurochemical lesion of vlPAG were significantly higher than that in the nonlesion group (Wang et al., 2019). These experimental results indicate that the vlPAG dysfunction might be involved in the formation of the comorbid migraine‐type headaches in epilepsy.

Brain‐derived neurotrophic factor (BDNF) is a major member of the neurotrophic family that is widely expressed in both central and peripheral nervous system (Du, 2008; Gordon, 2009). It has been demonstrated that BDNF‐secreting neurons in vlPAG not only helped to regulate neuronal survival during growth, differentiation, neurotransmission, and synaptic plasticity (Casarotto et al., 2010; Jiang et al., 2016; Yang et al., 2020), but are also involved in descending inhibition and descending facilitation by means of projections to rostral ventralmedial medulla (RVM) (Guo et al., 2006). The effects of BDNF are mediated by binding to its high‐affinity TrkB receptor and subsequent activation of downstream signaling conduction pathways (Huang & Reichardt, 2003). In experimental models, injection of low doses of BDNF enhanced pain sensitivity, while injection of high doses induced an effective analgesia (Merighi et al., 2008). With specific reference to chronic epilepsy, supplying of BDNF to the lesioned hippocampus facilitates “good” neurogenesis, reversing neuronal damage and possibly preventing seizures (Gu et al., 2017; Bovolenta et al., 2010; Paradiso et al., 2009). However, BDNF‐based therapeutic approaches for epilepsy are complicated. The opposite view suggested that intrahippocampal infusion or transgenic overexpression of BDNF increased seizure susceptibility or severity (McNamara & Scharfman, 2012; Weidner et al., 2011). These variable results may due to multiple factors, including the diverse animal models of epilepsy, the period of BDNF therapy in the history of epilepsy, the method of delivery, and subcellular localization (Greenberg et al., 2009; Iughetti et al., 2018; Tongiorgi et al., 2006).

Because of the complexity of epilepsy–migraine comorbidity, it is still unclear whether and how the overexpression of BDNF in PAG region contributes to modulate the comorbidities behavior. The aim of the current study was to analyze the antiepileptic and analgesic effects of BDNF–TrkB signaling pathway in PAG of migraine comorbid epilepsy.

## 2 MATERIALS AND METHODS

### 2.1 Ethics statement

This study was conducted in strict accordance with the Guidelines for the Use of Experimental Animals of Anhui Medical University of China. The experimental procedures were approved by the Committee on the Ethics of Animal Experiments of The First Affiliated Hospital of Anhui Medical University, China. Efforts were made to minimize animals use and suffering.

### 2.2 Animals

Adult male Sprague‐Dawley (280–300 g of weight) rats were used in the study. Four rats were housed in each standard polycarbonate cage at a constant temperature (25 ± 1°C) in a humidity‐controlled room (50 ± 5%) on a 12 h light/dark cycle (lights on 07:00–19:00 h), with free access to food and water. The rats were habituated in the experimental room for at least one week prior to the experiment.

### 2.3 Group division and models establishment

Rats were divided into 4 groups. Control group: Normal rats were used for control group. Epilepsy group: Rats were injected with freshly prepared lithium chloride solution (127 mg/kg, i.p., Cayman, USA). After 24 h, rats were pretreated with scopolamine methylbromide (1 mg/kg, i.p., Sigma, USA) to prevent activation of peripheral cholinergic receptors by pilocarpine. About 30 min later, rats were injected with pilocarpine (340 mg/kg, i.p., Cayman, USA) freshly diluted in 0.9% saline to induce status epilepticus (SE). If induced failure for some rats, pilocarpine solution (10 mg/kg, i.p.) needs to be re‐injected every 15 min (Glien et al., 2001). Migraine group: As previously described (Pradhan et al., 2014), rats were injected with nitroglycerin (NTG) (Beijing Yimin Pharmaceutical Co., Ltd; China) freshly diluted in 0.9% saline to a dose of 9.0 mg/kg every 2 days over 10 days (5 i.p. days total) as for the migraine group. Comorbid group: According to Fan et al.’s (2017) modeling method and then slightly improved, rats were induced to the epileptic model via pilocarpine injection, referring to the scheme of the second group, the model of chronic migraine was induced by injecting NTG; after one week, repeated NTG was administered to induce migraine‐like attacks.

**FIGURE 1:**
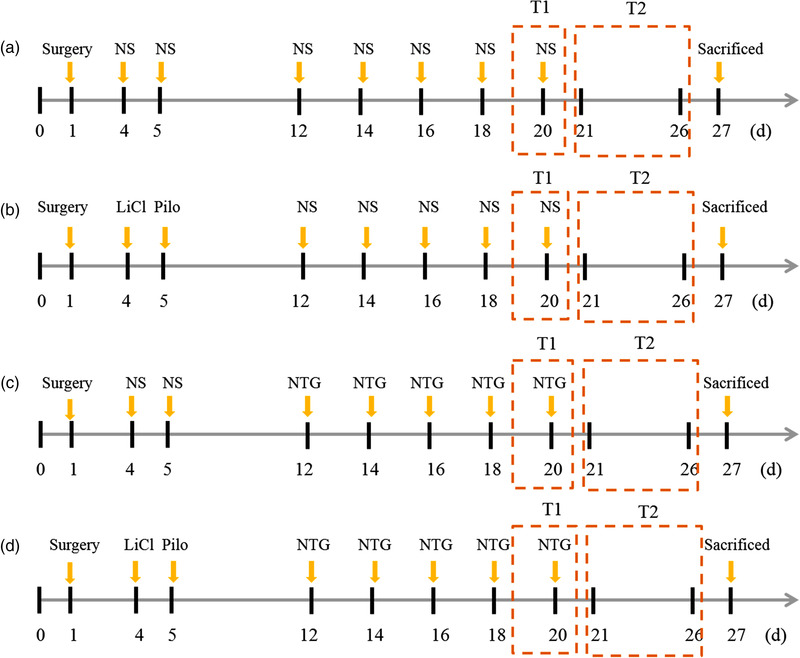
Experimental procedure. Pretreatment with AAV vector or NS: Microinjection of AAV vector and NS by surgery prior to modeling in each group. Status epilepticus (SE) was induced by i.p. injection of lithium chloride and pilocarpine at 4 and 5 days, respectively, in both epilepsy group (B) and comorbid group (D), while injecting the equal NS in the control group (A) and migraine group (C). Recurrent migraine‐like attacks were induced by i.p. injection of nitroglycerin (NTG) q.o.d. within 12–20 days in both C and D groups, while the equal NS in A and B group. T1: test 1, head scratching count and pain sensitivity test; T2: test 2, Electroencephalogram (EEG) implantation and detection. The rats were sacrificed and midbrain, high cervical spinal cord, trigeminal ganglion were removed for HE, TUNEL, immunohistochemical staining

The detailed experimental process is shown in Figure 1. In brief, adeno‐associated virus (AAV)‐BDNF, AAV and normal saline (NS) were microinjected into vlPAG, respectively, before modeling. Epileptogenic agent was used to induce SE in both the epilepsy (B) and comorbidity group (D) at 4 and 5 days, while the equal volume of normal saline was given in the migraine (C) and control group (A). NTG was repeatedly injected to induce migraine attacks in both the migraine and comorbidity group at 12, 14, 16, 18, and 20 days, while the equal volume of normal saline (NA) was injected into the epilepsy and control groups at the same time point. Pain sensitivity test was performed 1 h after NTG injection at 20 days and EEG implantation and monitoring were performed from 21 to 26 days. After behavioral test, the samples were collected at 27 days.

### 2.4 Intra‐BDNF stereotaxic microinjections

Rats were anesthetized with 2% pentobarbital sodium (80 mg/kg) and fixed the brain on the stereotactic apparatus with a microinjection syringe (RWD Life Science, China); then sheared the hairs, disinfected with 1% iodophor, and made a midline incision in the skull skin. After adjusting bregma and posterior fontanelle, two holes of left‐right were drilled and microinjection syringe was vertically introduced according to the following coordinates of vlPAG based on Paxinos and Watson's rat brain in the stereotactic coordinate atlas: 7.8 mm caudal to Bregma, 0.8 mm beside the midline, and 6.0 mm ventral to the surface of the cranium (Yang et al., 2018). Rats in the AAV–BDNF subgroups were slowly injected with a AAV‐mediated BDNF using microinjection syringe; rats in BDNF blank virus subgroup were slowly injected with the blank AAV vector; and rats in NS subgroup were injected with equal NS. After the operation, the rats were reared in single cage and recovered for 3 days. Then, rats were randomly selected from each treated group and separated into the following groups: Epilepsy group, migraine group, comorbidity group, and control group.

### 2.5 Behavioral tests

As shown in Figure 1, basal responses of thermal hyperalgesia, including paw withdrawal latency (PWL), head grooming and scratching, were considered as indexes of spontaneous migraine‐like episodes (Chanda et al., 2013) and were examined at 20 days. The frequency and duration of spontaneous recurrent seizures were recorded from 21 to 26 days.

### 2.6 Thermal pain sensitivity

Thermal pain sensitivity was assessed by measuring PWL in response to heat stimuli according to the protocol given by previous reports (Chen et al., 2006; Junger & Sorkin, 2000). Rats were placed on the surface of a 2‐mm‐thick glass plate covered with transparent plexiglass box (20 × 20 × 25 cm^3^). The plantar surface of the right hind paw was thermally stimulated by a radiant heat stimulator (BME410A, Institute of Biological Medicine, Academy of Medical Science, China) through a glass plate. The PWL was defined as the time from the onset of the heat stimulus to the occurrence of a hind paw lifted or licked. A cut off of 20 s was used to avoid tissue injury. Three stimuli were repeated for the hind paw per rat with an interval of 5 min between consecutive stimuli. The mean of the three observations was calculated as the average PWL.

### 2.7 Head scratching

Rats were placed in the single pet cages to habituate for 20 min at 20 days. The amount of head grooming and scratching was recorded by a video camera, and the total monitoring time for each rat was 30 min.

### 2.8 EEG recording

Rats were fixed in the stereotaxic instrument and anesthetized with 3.5% isoflurane. The recording electrode was implanted into the right cingulated gyrus by stereotactic technique using the coordinates as follows: 1.0 mm posterior to the anterior fontanelle, 0.4 mm lateral, and 2.0 mm subdural. A stainless steel wire was placed into the coordinates at 11.0 mm posterior to bregma (cerebellum) and is the grounding electrode. All the electrodes were assembly fixed to the skull using stainless steel screws and dental cement before the scalp was sutured. One week after electrode implantation, EEG recording was performed using a multichannel physiological signal recording system (MP150, Biopac, USA). Each recording lasted for 1 week and 3 times in total for 3 months. Seizure frequency was assessed with mean seizure events per week, and mean duration of one major seizure event as seizure duration. The amplitude and frequency of EEG were performed and analyzed by Sleepsign Software ( Kissei Comte co. LTD, Japan).

Experimental drug intervention and behavioral detection were independently performed by two researchers (LW and LL F).

### 2.9 Tissue preparations

After the last behavioral test, rats were anesthetized with 2% pentobarbital sodium (80 mg/kg) intraperitoneally, and perfused through the ascending aorta with 250 ml of 0.9% physiological saline followed by 200 ml of 4% paraformaldehyde. The brainstem with vlPAG tissue, the high‐level spinal cord with TNC tissue, and the trigeminal ganglion (TG) were removed and postfixed overnight for hematoxylin‐eosin (HE), TdT‐mediated dUTP Nick‐End Labeling (TUNEL), immumohistochemical staining (IHC), and immunofluorescence (IF) detection.

### 2.10 Hematoxylin‐eosin (HE) staining

After deparaffinization and gradient dehydration, the midbrain slides were stained with hematoxylin and eosin, and then mounted with Permount. Each slide (5 μm thickness) was examined and photographed under a photon microscope (DM6 B, Leica, Corporation, Germany).

### 2.11 TUNEL staining

**FIGURE 2:**
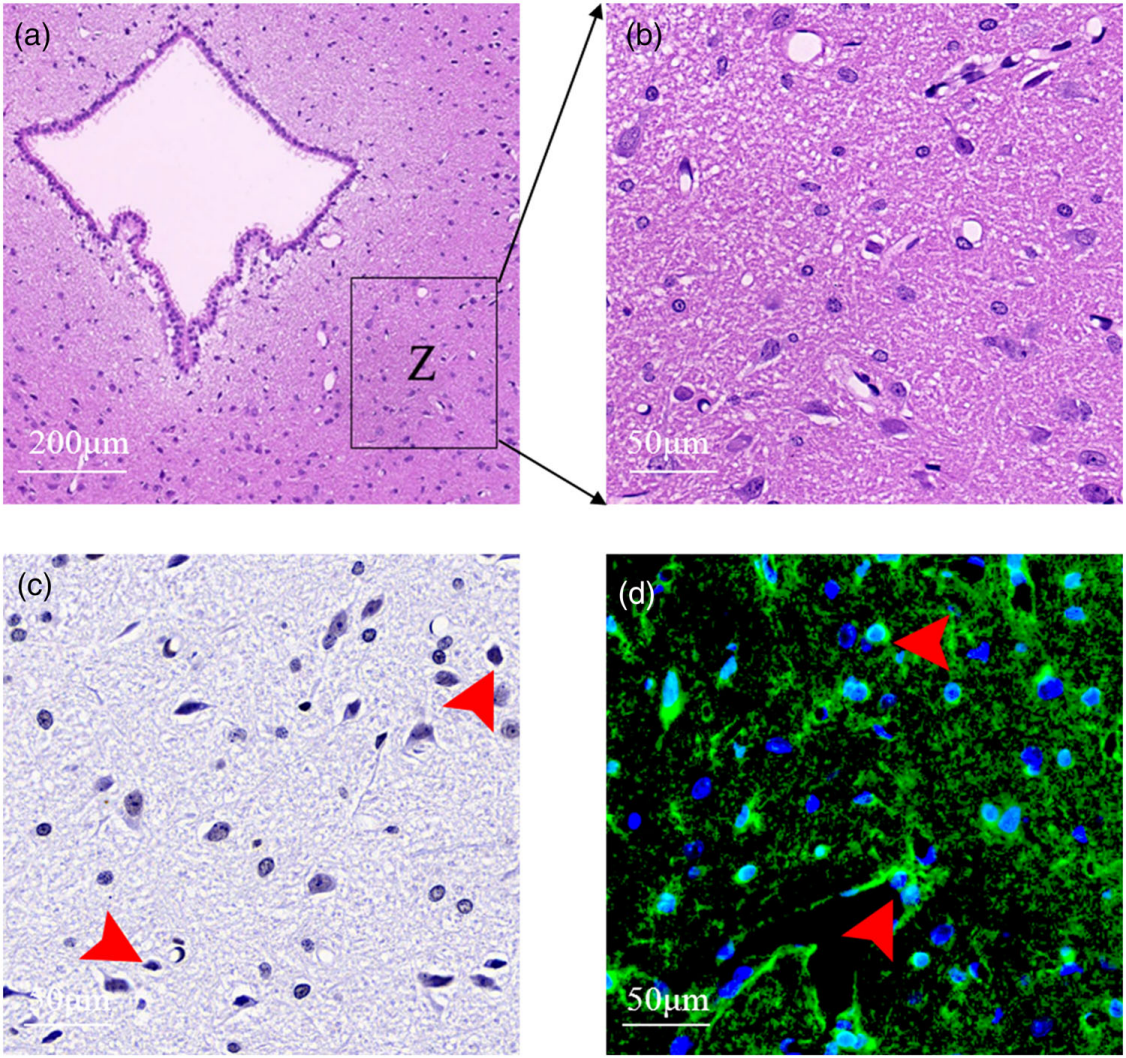
Graphic of the vlPAG area for cell counting and immunofluorescence intensity determination. Image B is the enlarged region of frame Z in image A with ×200 magnification, which was the area for cell counting and immunofluorescence intensity assay in HE, TUNEL and immunofluorescence staining. Image C shows the results of TUNEL staining in the same specimen as B. Image C shows the results of TUNEL staining in the same specimen as B. TUNEL‐positive cells are irregular with dense dark purple nuclei (red arrows). Image D represents the results of BDNF immunofluorescence staining in the same specimen as B. Red arrows indicate cells expressing BDNF

TUNEL staining was performed strictly following the instructions of apoptotic kit (Roche Ltd, USA). Briefly, each slice (5 μm thickness) was dewaxed and ethanol‐rehydrated, and then blocked with 3% H_2_O_2_ (ZSGB‐Bio, China) at 37°C for 30 min. After three PBS washes, slices were placed in 0.01 M citrate acid buffer (PH 6.0) and irradiated with microwave for 5 min. After being naturally cooled at room temperature, the slices were incubated with TUNEL solution (50 μl) at 37°C for 2 h. Then, following trice PBS washes, slices were incubated with Converter‐POD (50 μl) at 37°C for 30 min. After trice PBS washes, sections were incubated with 3,3′‐diaminodbenzidine (DAB) chromogenic solution (ZSGB‐Bio, China) at room temperature for 3 min. For the negative control, slices were only dropped labeled solution instead of TUNEL solution. TUNEL‐positive cells were counted in 1 out of 20 slices across the vlPAG, that is, 5 slices per rat. In each section, two symmetric counting frames were placed at the vlPAG aqueduct on images of ×200 magnification according to the Rat Brain in Stereotaxic Coordinates (Figure 2B,C). Counting was independently employed by two investigators (LW and LL F), and two counts were averaged to obtain a singer number per frame.

### 2.12 Immunohistochemical detection

Consecutive sections of 4% paraformaldehyde‐fixed and paraffin‐embedded tissues were cut at a thickness of 4 μm, adhered to antislip slides, and then dried overnight at 50°C. After dewaxing and rehydrating, the slides were immersed into citrate buffer (pH = 6.0), which was heated to 100°C for 15 min, cooled at room temperature for 10 min, and then blocked with a 5% goat serum for 5 min. After being washed 3 times with PBS at 5‐min intervals, the slides with spinal cord tissue and TG were respectively incubated with the rabbit anti‐c‐Fos (1:100, BS 1130, Bioworld) and rabbit anti‐CGRP (1:100, ab47027, Abcam) primary antibody overnight at 4°C. After being rinsed 3 times with PBS at 2‐min intervals, slides were incubated with goat antirabbit IgG (1:500) for 30 min, followed by incubation of DAB. Finally, section slides were counterstained with hematoxylin (H9627, Merck) for 3 min, rinsed with PBS for 3 times, and then sealed with Permount. Specific 500 × 200 μm^2^ regions (Figure 10A) on both sides of the TNC were captured under 200× magnification with a light microscope (DM6 B, Leica, Germany). Both the c‐Fos and CGRP immune positive cells were distinguished by brown granule labeling. The number of positive cells were counted using Image Pro Plus 6.0 Software by a professional who was blinded to the experiment.

### 2.13 Immunofluorescence staining

Paraffin‐embedded midbrain tissues were pretreated in the same way as immunohistochemistry. After being blocked with a goat serum, slides were incubated with rabbit anti‐BDNF primary antibody (1:500, ab108319, Abcam) and rabbit anti‐TrkB primary antibody (1:500, GB11295‐2, Servicebio), respectively, at 4°C overnight. After 3 washes with 0.01 M PBST at 2 min intervals, slides were incubated with goat antirabbit Alexa 488 (1:400, GB25303, Servicebio) for BDNF and Cy3 conjugated Goat Anti‐Rabbit IgG (GB21303, 1:300, Servicebio) for TrkB under darkness for 1.5 h. After staining, slides were washed with PBS and counterstained with 0.00001% DAPI (Santa Cruz, USA) for 15 min. Slides were again rinsed 3 times with PBS and then sealed in antifluorescent quenching medium. The expressions of BDNF and TrkB in vlPAG positive cells were quantitatively detected by immunofluorescence staining using Image Pro Plus 6.0 Software. In each section, two symmetric counting frames were placed ventrolaterally to the midbrain aqueduct on images of ×200 magnification basing on the Rat Brain in Stereotaxic Coordinates (Figure 2D). By manually converting the captured image and setting the threshold, any background signal was set to 0, and background signals were completely removed from the calculation. Three frames of images were randomly set to cover the vlPAG region to be analyzed, and the gray values were measured. The measurements were made on 5 separate sections per rat, in 7 rats per group.

### 2.14 Statistical analysis

All data were tested for normal distribution expressed as mean ± standard error (M ± SEM). Data analysis was examined by analysis of variance with SPSS 16.0 software. A Tukey's post‐hoc correlation was conducted after variance analysis. *p *< 0.05 was considered as significant difference.

## 3 RESULTS

### 3.1 Behavioral tests

#### 3.1.1 The effects of AAV–BDNF injection on pain sensitivity and head scratching

After NS, AAV, and AAV–BDNF intracranial injection for 3 weeks, the changes of PWL and the number of head scratching in each group were detected.

**FIGURE 3:**
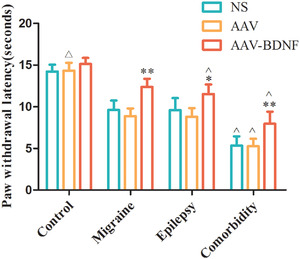
PWL changes in each treatment group. PWL was not significantly different among the control subgroups (*^△^p *> 0.05). AAV–BDNF injected rats in both the monomorphic and comorbidity groups exhibited significantly lengthened PWL compared to NS or AAV vector‐injected animals. (*p < 0.05, ˆ*p *< 0.05). In the comorbidity groups, three subgroups exhibited significantly lengthened PWL than that of the corresponding monomorphic and control subgroups (ˆ*p *< 0.05). Data are expressed as Mean ± SEM. A One‐way ANOVA was performed. n = 7 per group

PWL test results showed that there was no significant difference among subgroups in the control group (Figure 3, ^△^ *p *> 0.05). The AAV–BDNF injected rats both in the monomorphic and comorbidity groups exhibited significantly lengthened PWL compared to NS or AAV vector‐injected animals. (Figure 3, *p < 0.05, **p < 0.01). In the comorbidity group, the PWLs of the three subgroups were higher than that of the corresponding subgroups in both monomorphic and control groups (Figure 3, ˆ*p *< 0.01).

**FIGURE 4:**
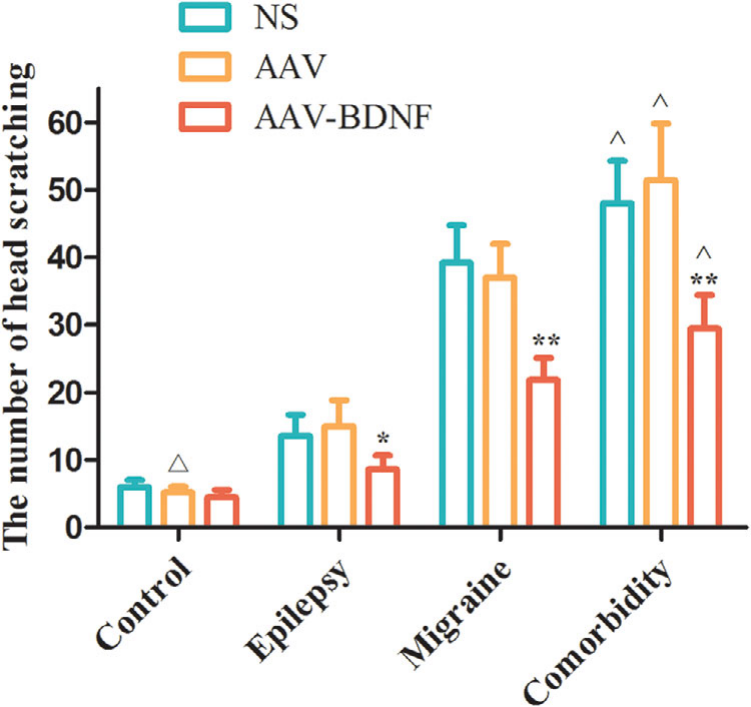
The change of scratching frequency in each treatment group. The subgroup rats in the control group showed less head scratching frequency with no significant difference (*^△^p *> 0.05). AAV–BDNF injected rats in both the monomorphic and comorbidity groups exhibited significantly increased number of head scratching compared to NS or AAV vector‐injected animals (*p < 0.05, **p < 0.01). In the comorbidity group, the number of head scratching of the three subgroups were higher than that of the corresponding subgroups in both monomorphic and control groups (ˆ*p *< 0.01). Data are expressed as Mean ± SEM. A One‐way ANOVA was performed. *n* = 7 per group

The subgroup rats in the control group showed less head‐scratching frequency with no significant difference (Figure 4, *^△^p *> 0.05). The AAV–BDNF injected rats in both the monomorphic and comorbidity groups exhibited significantly increased number of head scratching compared to NS or AAV vector‐injected animals (Figure 4, *p < 0.05, **p < 0.01). In the comorbidity group, the number of head scratching of the three subgroups were higher than that of the corresponding subgroups in both monomorphic and control groups (Figure 4, ˆ*p *< 0.01).

#### 3.1.2 The effects of AAV–BDNF injection on seizure episodes

**FIGURE 5:**
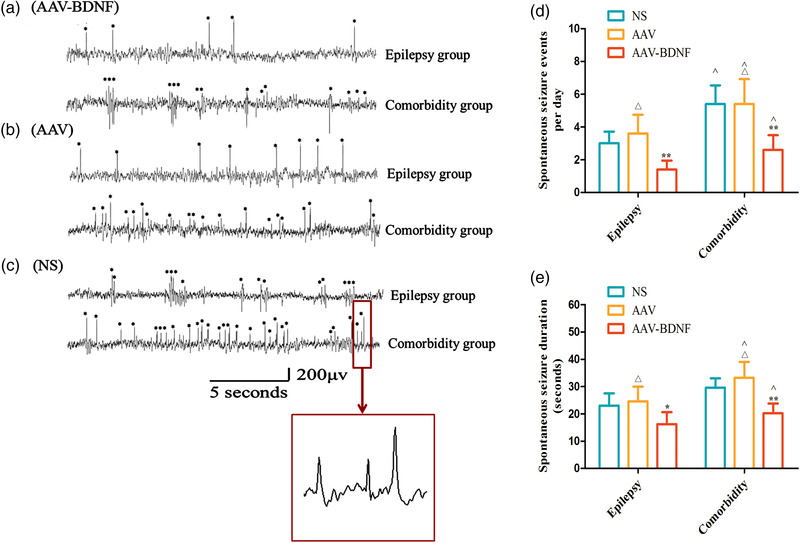
Overexpression of BDNF in vlPAG alleviates the Interepileptic peak discharges and spontaneous seizure events in epilepsy–migraine comorbid rats. Typical EEG recordings from epilepsy group and epilepsy–migraine comorbid group with AAV–BDNF (A), AAV (B), and NS (C) intervention. Epileptic peak in EEG is characterized by sharp peak and high amplitude (asterisks on A–C), and the typical peak is demonstrated in big square box enlarged from that in small square frame (C). (D, E) Statistical analysis showed significantly reduced the spontaneous seizures and seizure duration in the AAV–BDNF injection subgroup than those in the AAV or NS injection subgroups (*p < 0.05, **p < 0.01). There was no significant difference between AAV and NS subgroups (*^△^p *> 0.05). In addition, under the same intervention, the spontaneous seizures and seizure duration in the comorbidity group were higher than those in the epilepsy group (ˆ*p *< 0.05). Data was Mean ± SEM. A One‐way ANOVA was conducted. *n* = 7 per group

At the end of the pain behavioral test, rats in the epilepsy and comorbidity groups were implanted with EEG electrodes and monitored. Epileptiform interictal peaks on EEG displayed sharp peak and high amplitude, which were labeled with asterisks (Figure 5A–C). Data monitoring detected a significant reduction of spontaneous seizures and seizure duration in the AAV–BDNF injection subgroup than those in the AAV or NS injection subgroups (Figure 5D,E, *p < 0.05, **p < 0.01). There was no significant difference between AAV and NS subgroups (Figure 5D,E, *^△^p *> 0.05). In addition, under the same intervention conditions, the spontaneous seizures and seizure duration in the comorbidity group were higher than those in the epilepsy group (Figure 5D,E, ˆ*p *< 0.05).

#### 3.1.3 Histological changes in vlPAG after AAV–BDNF intervention

**FIGURE 6:**
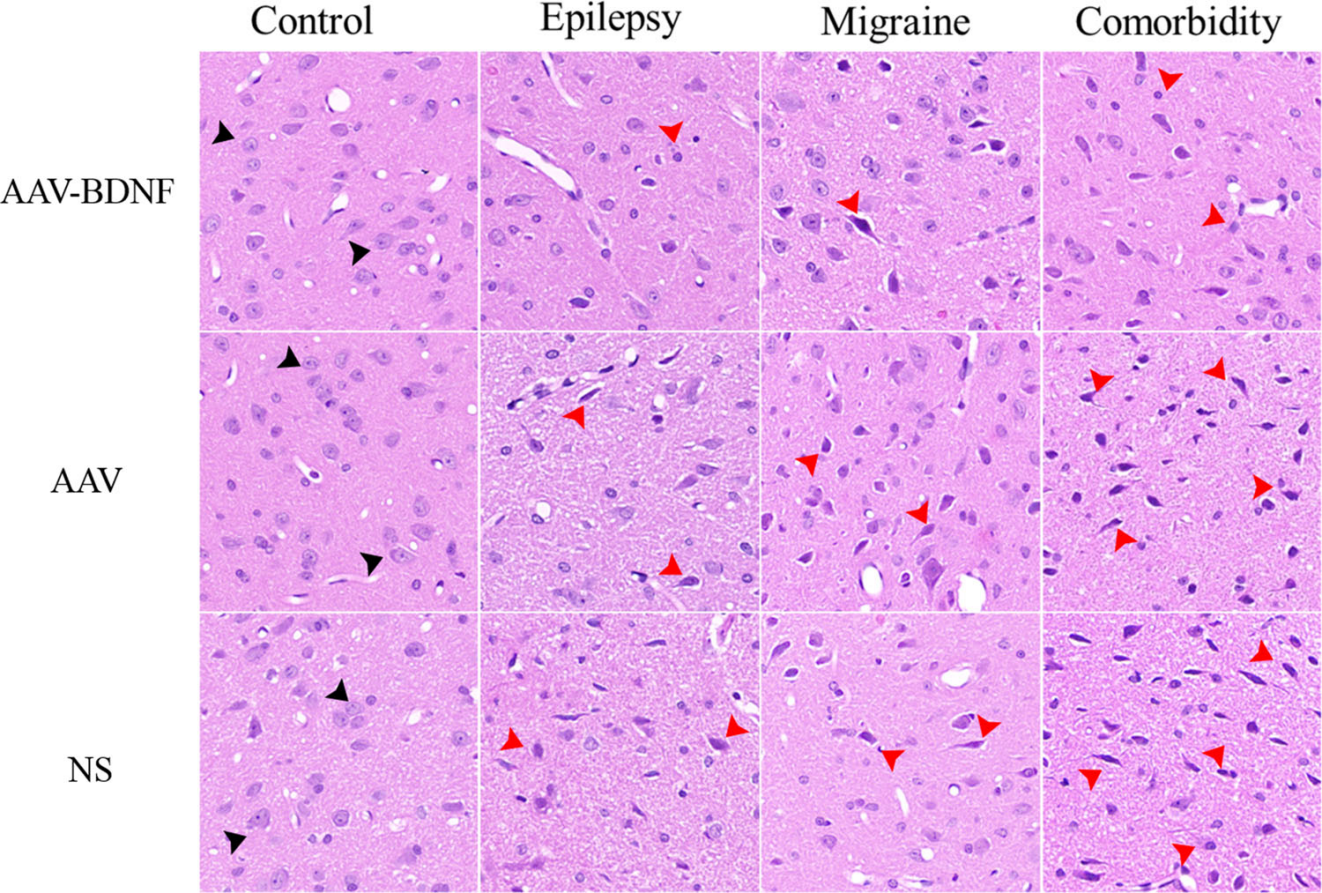
HE staining of the vlPAG area. More normal‐shaped neurons (black arrows) were observed in the AAV–BDNF subgroups as compared to those in AAV or NS subgroups in all model rats. The damaged neurons showed deeply staining and the injured neurons were deeply stained and shrunken spindle cells, especially in comorbidity group (red arrows). *n* = 5 per group. Scale bar = 50 μm

In the control group, HE staining showed that the neurons in vlPAG region were regularly round or oval with clear cytoplasm. Round, transparent, and hyperchromatic nucleus was displayed in the cytoplasm (black arrows, Figure 6). The damaged neurons showed strong staining and the injured neurons were also strongly stained and had shrunken spindle cells, especially in the comorbidity group (red arrows in Figure 6). In addition, the pathological lesions in both the AAV‐ and NS‐treated subgroups were more obvious compared with those in the AAV–BDNF‐treated subgroup (Figure 6). This indicates that overexpression of BDNF in vlPAG region can alleviate pathological damage in chronic phase of each model group.

#### 3.1.4 Cellular apoptosis in vlPAG after AAV–BDNF intervention

**FIGURE 7:**
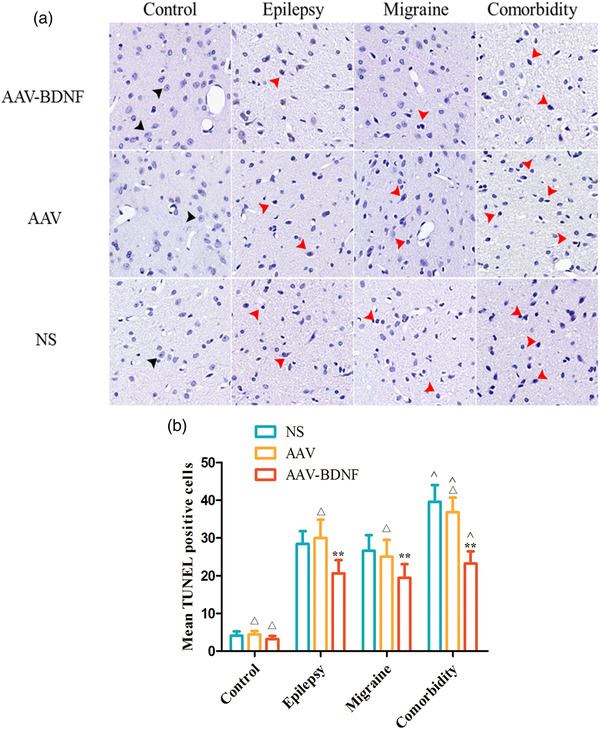
BDNF overexpression reduces neuronal cell loss. (A) The number of TUNEL‐positive cells in model groups was significantly increased than those in the control group, and they presented irregular shape with condensed purple brown nucleus (red arrows). (B) Few TUNEL‐positive cells were observed between AAV and NS subgroups with no significant difference (*^△^p *> 0.05). The average number of TUNEL‐positive cells in AAV and NS injection subgroups was significantly higher than that in AAV–BDNF injection subgroups (*p < 0.05, **p < 0.01). In addition, in the comorbidity subgroup, the average number of TUNEL‐positive cells was significantly higher than that of the corresponding monomorphic subgroup (ˆ*p *< 0.05). Data was Mean ± SEM. A one‐way ANOVA was performed. *n* = 7 per group. Scale bar = 50 μm

In the control group, few TUNEL‐positive cells were observed between AAV and NS subgroups with no significant difference (Figure 7A,B, *^△^p *> 0.05). The number of TUNEL‐positive cells in model groups were significantly increased than those in the control group, and they presented irregular shape with condensed purple brown nucleus (red arrows in Figure 6A). The average number of TUNEL positive cells in AAV and NS injection subgroups was significantly higher than that in AAV–BDNF injection subgroups (Figure 7B, *p < 0.05, **p < 0.01). In addition, in the comorbidity subgroup, the average number of TUNEL‐positive cells was significantly higher than that of the corresponding monomorphic subgroup (Figure 7B, ˆ*p *< 0.05).

#### 3.1.5 Expression levels of BDNF and TrkB proteins in vlPAG

**FIGURE 8:**
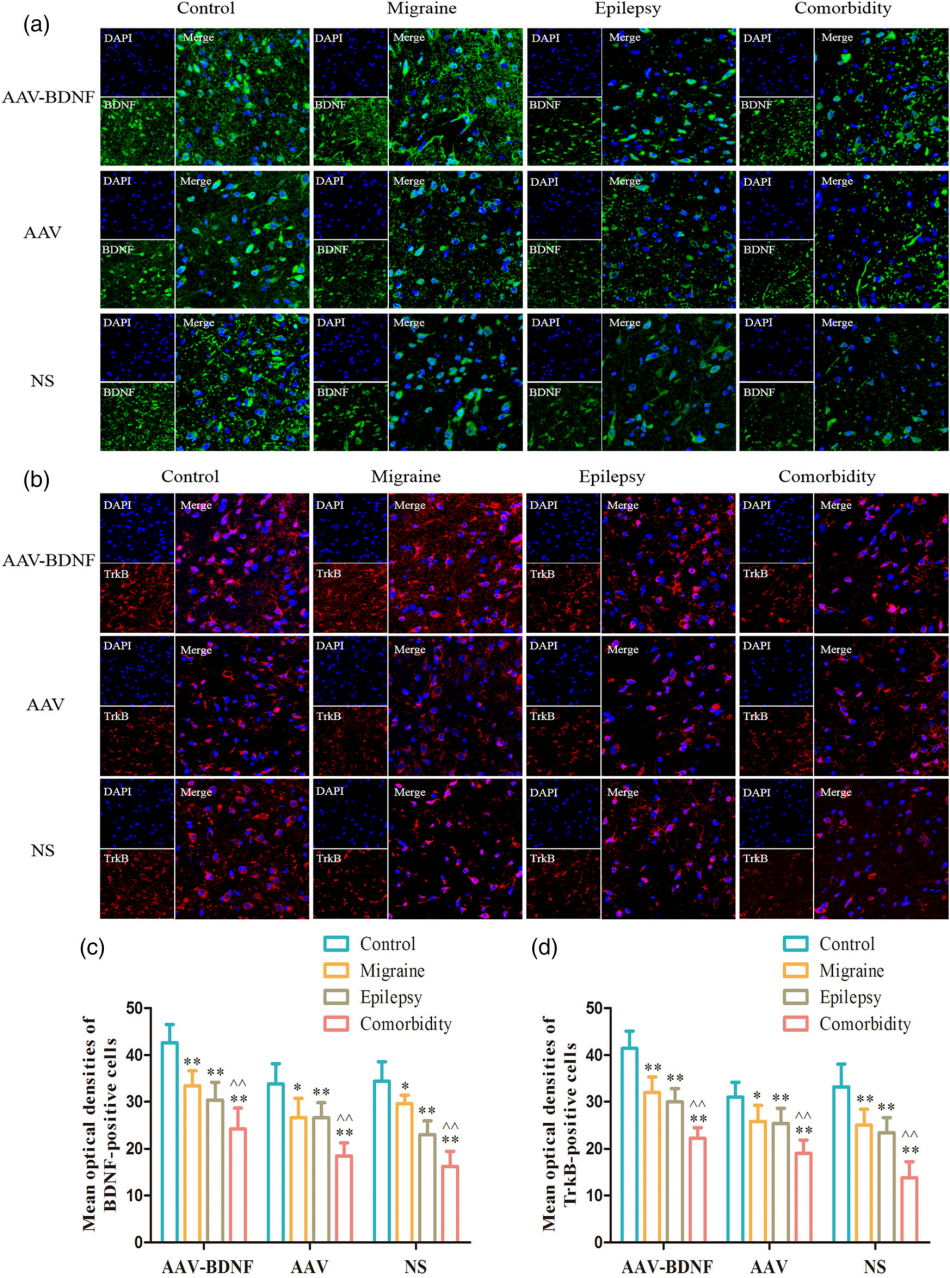
Optical densities of BDNF‐ and TrkB‐positive neurons in vlPAG. (A,B) Immunofluorescence staining showed that BDNF and TrkB puncta appeared uniformly along the cytomembrane in all subgroups. (C,D) The average optical densities of BDNF‐ and TrkB‐positive cells in all model subgroups were significantly lower than those in the corresponding control subgroups (*p < 0.05, **p < 0.01). In addition, the mean optical densities of BDNF‐ and TrkB‐positive cells in the comorbidity subgroup were significantly lower than that of the corresponding monomorphic subgroup (ˆ*p *< 0.05, ˆˆ*p *< 0.01). Scale bar (upper and lower panel) = 100 μm, Scale bar (middle panel) = 50 μm. Data was Mean ± SEM. A one‐way ANOVA was performed. n = 7 per group

Immunofluorescence staining showed that BDNF and TrkB puncta appeared uniformly along the cytomembrane in all subgroups (Figure 8A,B). The mean optical densities of BDNF‐ and TrkB‐ positive cells in all model subgroups were significantly lower than those in the corresponding control subgroups (Figure 8C,D, *p < 0.05, **p < 0.01). In addition, the average optical densities of BDNF‐ and TrkB‐positive cells in the comorbidity subgroup were significantly lower than that of the corresponding monomorphic subgroup (Figure 8C,D, ˆ*p *< 0.05, ˆˆ*p *< 0.01).

#### 3.1.6 The effects of AAV–BDNF injection on c‐Fos and CGRP immunoreactive cells in TNC and TG

**FIGURE 9:**
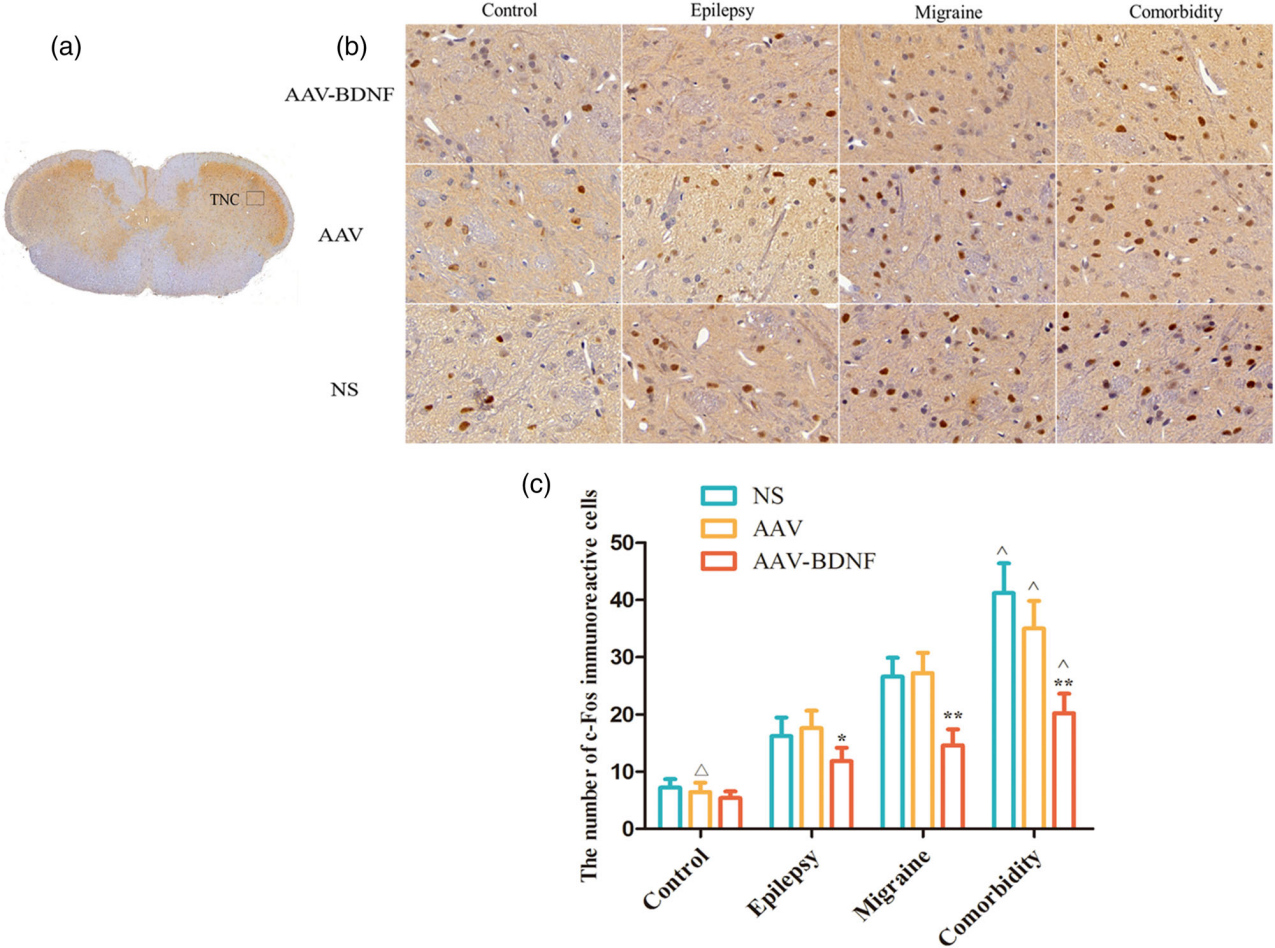
BDNF treatment reduces c‐Fos immunoreactive cells in TNC. (A) c‐Fos immunoreactive cells were investigated in TNC (black rectangle). (B) Immunohistochemical staining showed that c‐Fos puncta were presented in the brownish yellow or nigger‐brown nucleus. (C) The expression of c‐Fos immunoreactive neurons was less among control subgroups with no significant difference (*^△^p *> 0.05). In all model groups, the average c‐Fos immunoreactive cells in AAV–BDNF injection subgroups were significantly lower than those in the corresponding AAV or NS injection subgroups (*p < 0.05, **p < 0.01). The average c‐Fos immunoreactive cells in theomorbidity subgroups were significantly higher than that of the corresponding monomorphic subgroups (ˆ*p *< 0.05). Data was Mean ± SEM. A one‐way ANOVA was performed. *n* = 7 per group. Scale bar = 50 μm

**FIGURE 10:**
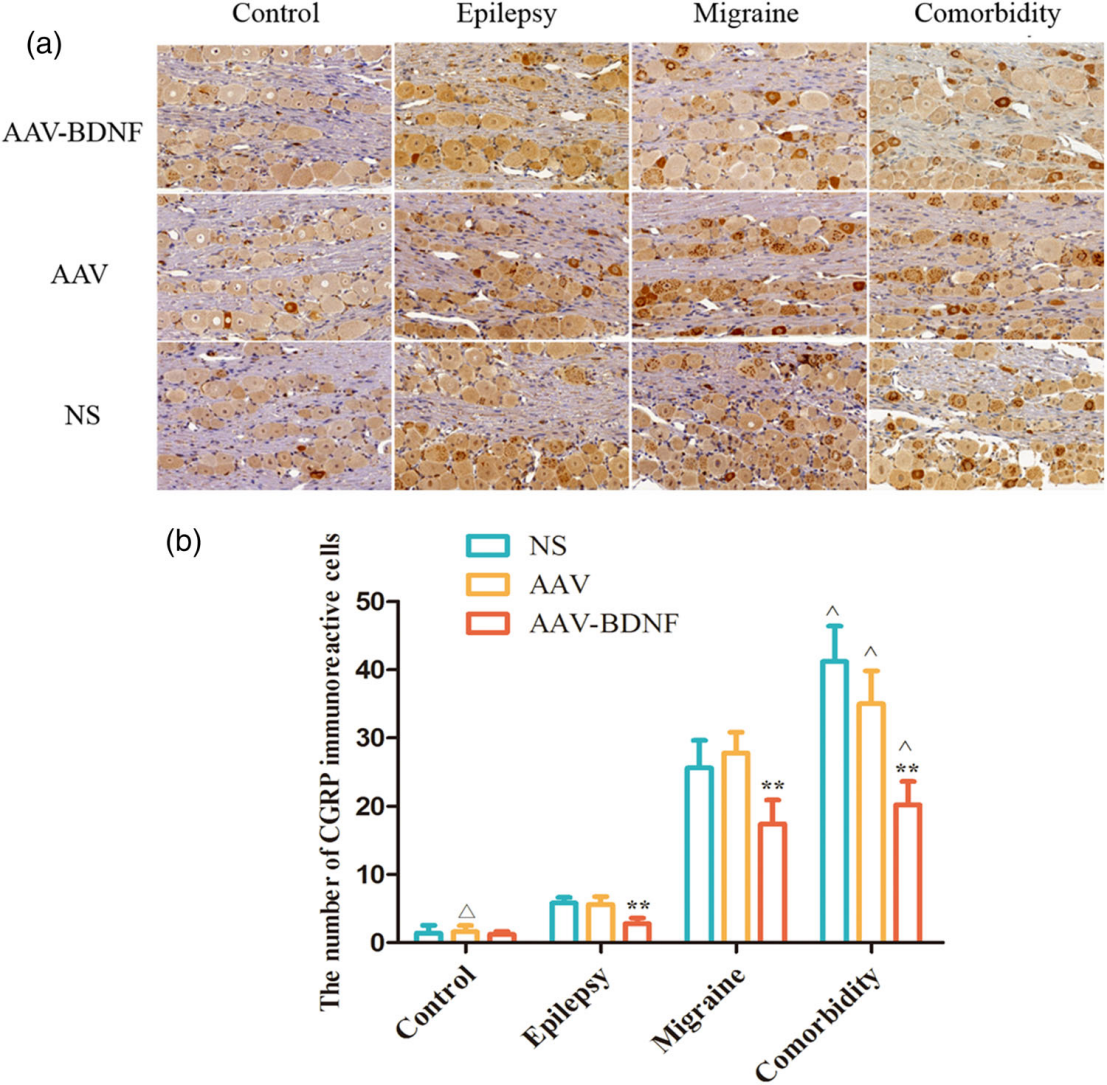
BDNF treatment reduces CGRP immunoreactive cells in TG. (A) Immunohistochemical staining showed that CGRP puncta were presented in the brownish yellow or nigger‐brown nucleus. (B) The expression of CGRP immunoreactive neurons was less among control subgroups with no significant difference (*^△^p *> 0.05). In all model groups, the average CGRP immunoreactive cells in AAV–BDNF injection subgroups were significantly lower than those in the corresponding AAV or NS injection subgroups (*p < 0.05, **p < 0.01). The average CGRP immunoreactive cells in theomorbidity subgroups were significantly higher than that of the corresponding monomorphic subgroups (ˆ*p *< 0.05). Data was Mean ± SEM. A One‐Way ANOVA was performed. *n* = 7 per group. Scale bar = 50 μm

c‐Fos immunoreactive cells were investigated in TNC (black rectangle) (Figure 9A). Immunohistochemical staining showed that c‐Fos and CGRP puncta were presented in the brownish yellow nucleus and nigger‐brown cytoplasm, respectively. (Figures 9B and 10A). The expressions of c‐Fos and CGRP immunoreactive neurons were less among control subgroups with no significant difference (Figures 9C and 10B, *^△^p *> 0.05). In all model groups, the average c‐Fos and CGRP immunoreactive cells in AAV–BDNF injection subgroups were significantly lower than those in the corresponding AAV or NS injection subgroups (Figures 9C and 10B, *p < 0.05, **p < 0.01). In addition, the average c‐Fos and CGRP immunoreactive cells in the comorbidity subgroups were significantly higher than that of the corresponding monomorphic subgroups (Figures 9C and 10B, ˆ*p *< 0.05).

## 4 DISCUSSION

Previous studies demonstrated that comorbid migraine‐type headaches have negative impact on epilepsy outcomes, including intractable condition incidence and drug‐resistant epilepsy formation (Dragoumi et al., 2013). The establishment of an effective animal model of comorbid epilepsy and migraine is an important tool for elucidating the comorbid mechanism and developing new therapies. In the current study, we successfully induced the epilepsy–migraine comorbid rat model on the basis of Fan et al.’s study (Fan et al., 2017). CGRP is mainly expressed in both the TG and vascular system, which plays an important regulatory role in the generation and maintenance of migraine, central sensitization, and allodynia, events characteristic of migraine pathology (Durham & Vause, 2010). Therefore, CGRP is recognized as a neurobiological marker of migraine (Edvinsson, 2019; Edvinsson & Warfvinge, 2019). In addition, c‐Fos is an immediate early gene, which is rapidly and transiently expressed in response to central pain pathway (Akerman et al., 2007). So c,‐Fos is also widely used as a common marker in the study of migraine (Mitsikostas & Sanchez del Rio, 2001; Park et al., 2014). Through animal behavior observation and related specific markers detection, the feasibility of establishing the comorbid model was verified. In addition, our present study found that BDNF overexpression in the vlPAG region highly significantly decreased the frequency and duration of spontaneous seizures as well as pain sensitivity in the epilepsy–migraine comorbid rat. This suggests that protective intervention for vlPAG may help to reduce the risk of nociceptive behavior caused by epileptic seizures.

vlPAG is the key nucleus of the descending pain inhibition system, and has a bidirectional effect on the regulation of pain perception (Keay & Bandler, 1998; Menant et al., 2016). Stimulation of the vlPAG promotes plastic changes during neuronal firing and analgesic effects in epileptic rats (Lam et al., 2010; Wang et al., 2019), while inhibition of vlPAG neurons leads to hyperalgesia (Bartsch et al., 2004). Apoptosis and changes of excitatory and inhibitory in vlPAG facilitated the occurrence of epileptogenesis and migraineogenesis (Samineni et al., 2017; Wang et al., 2019). This phenomenon is even more evident in our comorbid model. So, it is reasonable to assume that the vlPAG structure and dysfunction might be involved in the occurrence of the epilepsy–migraine comorbidity.

Neurotrophins and neurotrophin receptors, particularly BDNF and its receptor‐TrkB, lead to both epilepsy and migraine (Karatas et al., 2018; Martins et al., 2017; Simonato et al., 2006). Falcicchia et al. (2018) implanted encapsulated cell biodelivery devices filled with sustainably released BDNF transgenic human cells into the hippocampus of pilocarpine‐treated rats, which highly decreased the frequency of spontaneous seizures and improved cognitive performance. In the current study, AAV–BDNF injection of monopathy and comorbid group showed higher average optical densities of BDNF‐ and TrkB‐positive neurons, as well as higher total expression levels of BDNF and TrkB proteins in vlPAG compared to the AAV‐ or NS‐ treated groups. These were consistent with the results of tests for epileptic and migraine‐like behaviors, which indicates that BDNF–TrkB signaling may also have antiepileptogenic and analgesic effects in epilepsy comorbid with migraine. More relevant to the current findings, BDNF was found to reduce GABA_A_ receptor desensitization in the murine epileptic hippocampus during the chronic epileptic stage (Palma et al., 2005). However, in contrast with present finding, a herpes simplex vector (HSV)‐mediated injection of BDNF in the hippocampus of epileptic rats had no effect on the frequency or severity of the spontaneous seizure (Paradiso et al., 2009). The reason for the contrasting effects on seizures depends on the epileptic model, administration time, disease stage required for the experiment, and the TrkB activation or inhibition strategy (Greenberg et al., 2009).

In fact, neurons and astrocytes in vlPAG also express BDNF (Conner et al., 1997). In addition to participating in neural remodeling in epilepsy (Fernandez‐Garcia et al., 2020), BDNF secreting projection neurons in vlPAG can also enhance the expression of presynaptic of neuroactive substances such as CGRP, serotonin (5‐HT), and nitric oxide synthase (NOS) by binding presynaptic TrkB in the vlPAG‐RVM pathway to involve in the descending pain modulation (Yin et al., 2014). In the current study, the expression levels of relative markers in TNC and TG in each treatment subgroup were consistent with the corresponding behavioral changes, which also supports the previously mentioned view. Therefore, BDNF exerts a real antiepileptogenic and analgesic effect, and not merely a therapeutic effect.

Our results show that the expression levels of BDNF and TrkB in the comorbid group were lower than those in the mononuclear group under the same intervention conditions, which is also consistent with the results of behavioral tests. This indicates that the comorbid epilepsy and migraine shares common mechanisms including BNDF‐TrkB signaling pathway and neuroanatomical regions—vlPAG.

## 5 CONCLUSION

In summary, overexpression of BDNF in vlPAG decreased the severity of epileptic and migraine‐like events in comorbid rats, pointing to an antiseizure and analgesic effect via the activation of BDNF–TrkB signaling pathway. Despite uncertainty as to the exacted mechanisms underlying the generation of migraine in epilepsy, our findings support a strongly positive effect of supplementation of BDNF in the vlPAG, and also provide a foundation for further research on the mechanism and treatment of epilepsy comorbid migraine.

## AUTHOR CONTRIBUTIONS

Long Wang, Juan Zhang, Zi‐Ru Deng, and Lu‐Lan Fu carried out all vivo experiments and immunohistochemistry. Long Wang conducted the Western Blot. Long Wang, Jun‐Cang Wu and Yu Wang designed the current study and wrote the paper.

### PEER REVIEW

The peer review history for this article is available at https://publons.com/publon/10.1002/brb3.2594

## Data Availability

The datasets used and/or analyzed in this article are available from the corresponding author on reasonable request.

## References

[brb32594-bib-0001] Akerman, S. , Holland, P. R. , & Goadsby, P. J. (2007). Cannabinoid (CB1) receptor activation inhibits trigeminovascular neurons. The Journal of Pharmacology and Experimental Therapeutics, 320(1), 64–71. 17018694 10.1124/jpet.106.106971

[brb32594-bib-0002] Bartsch, T. , Knight, Y. E. , & Goadsby, P. J. (2004). Activation of 5‐HT(1B/1D) receptor in the periaqueductal gray inhibits nociception. Annals of Neurology, 56(3), 371–381. 15349864 10.1002/ana.20193

[brb32594-bib-0003] Bovolenta, R. , Zucchini, S. , Paradiso, B. , Rodi, D. , Merigo, F. , Navarro Mora, G. , Osculati, F. , Berto, E. , Marconi, P. , Marzola, A. , Febene, P. A. , & Simonato, M. (2010). Hippocampal FGF‐2 and BDNF overexpression attenuates epileptogenesis‐associated neuroinflammation and reduces spontaneous recurrent seizures. Journal of Neuroinflammation, 7, 81. 21087489 10.1186/1742-2094-7-81 PMC2993685

[brb32594-bib-0004] Casarotto, P. C. , de Bortoli, V. C. , Correa, F. M. , Resstel, L. B. , & Zangrossi, H. Jr. (2010). Panicolytic‐like effect of BDNF in the rat dorsal periaqueductal grey matter: The role of 5‐HT and GABA. The International Journal of Neuropsychopharmacology, 13(5), 573–582. 20047714 10.1017/S146114570999112X

[brb32594-bib-0005] Chanda, M. L. , Tuttle, A. H. , Baran, I. , Atlin, C. , Guindi, D. , Hathaway, G. , Israelian, N. , Levenstadt, J. , Low, D. , Macrae, L. , O'Shea, L. , Silver, A. , Zendegui, E. , Lenselink, A. M. , Spijker, S. , Ferrari, M. D. , van den Maagdenberg, A. M. J. M. , & Mogil, J. S. (2013). Behavioral evidence for photophobia and stress‐related ipsilateral head pain in transgenic Cacna1a mutant mice. Pain, 154(8), 1254–1262. 23673147 10.1016/j.pain.2013.03.038

[brb32594-bib-0006] Chen, Y. N. , Li, K. C. , Li, Z. , Shang, G. W. , Liu, D. N. , Lu, Z. M. , Zhang, J. W. , Ji, Y. H. , Gao, G. D. , & Chen, J. (2006). Effects of bee venom peptidergic components on rat pain‐related behaviors and inflammation. Neuroscience, 138(2), 631–640. 16446039 10.1016/j.neuroscience.2005.11.022

[brb32594-bib-0007] Conner, J. M. , Lauterborn, J. C. , Yan, Q. , Gall, C. M. , & Varon, S. (1997). Distribution of brain‐derived neurotrophic factor (BDNF) protein and mRNA in the normal adult rat CNS: Evidence for anterograde axonal transport. Journal of Neuroscience, 17(7), 2295–2313. 9065491 10.1523/JNEUROSCI.17-07-02295.1997 PMC6573520

[brb32594-bib-0008] de Freitas, R. L. , de Oliveira, R. C. , de Oliveira, R. , Paschoalin‐Maurin, T. , de Aguiar Correa, F. M. , & Coimbra, N. C. (2014). The role of dorsomedial and ventrolateral columns of the periaqueductal gray matter and in situ 5‐HT(2)A and 5‐HT(2)C serotonergic receptors in post‐ictal antinociception. Synapse, 68(1), 16–30. 23913301 10.1002/syn.21697

[brb32594-bib-0009] De Simone, R. , Ranieri, A. , Marano, E. , Beneduce, L. , Ripa, P. , Bilo, L. , Meo, R. , & Bonavita, V. (2007). Migraine and epilepsy: Clinical and pathophysiological relations. Neugological Sciences, 28(Suppl 2), S150–S155. 10.1007/s10072-007-0769-1 17508163

[brb32594-bib-0010] Dragoumi, P. , Tzetzi, O. , Vargiami, E. , Pavlou, E. , Krikonis, K. , Kontopoulos, E. , & Zafeiriou, D. I. (2013). Clinical course and seizure outcome of idiopathic childhood epilepsy: Determinants of early and long‐term prognosis. BMC Neurology, 13, 206. 24350775 10.1186/1471-2377-13-206 PMC3878358

[brb32594-bib-0011] Du, J. (2008). [The messengers from peripheral nervous system to central nervous system: Involvement of neurotrophins and cytokines in the mechanisms of acupuncture]. Zhen ci yan jiu = Acupuncture research, 33(1), 37–40. 18386643

[brb32594-bib-0012] Durham, P. L. , & Vause, C. V. (2010). Calcitonin gene‐related peptide (CGRP) receptor antagonists in the treatment of migraine. CNS Drugs, 24(7), 539–548. 20433208 10.2165/11534920-000000000-00000 PMC3138175

[brb32594-bib-0013] Edvinsson, L. (2019). Role of CGRP in migraine. Handbook of Experimental Pharmacology, 255, 121–130. 30725283 10.1007/164_2018_201

[brb32594-bib-0014] Edvinsson, L. , & Warfvinge, K. (2019). Recognizing the role of CGRP and CGRP receptors in migraine and its treatment. Cephalalgia : An International Journal of Headache, 39(3), 366–373. 29020807 10.1177/0333102417736900

[brb32594-bib-0015] Falcicchia, C. , Paolone, G. , Emerich, D. F. , Lovisari, F. , Bell, W. J. , Fradet, T. , Wahlberg, L. U. , & Simonato, M. (2018). Seizure‐Suppressant and Neuroprotective Effects of Encapsulated BDNF‐Producing Cells in a Rat Model of Temporal Lobe Epilepsy. Molecular therapy Methods & Clinical Development, 9, 211–224. 29766029 10.1016/j.omtm.2018.03.001 PMC5948312

[brb32594-bib-0016] Fan, S. , Xiao, Z. , Zhu, F. , He, X. , & Lu, Z. (2017). A new comorbidity model and the common pathological mechanisms of migraine and epilepsy. American Journal of Translational Research, 9(5), 2286–2295. 28559979 PMC5446511

[brb32594-bib-0017] Fernandez‐Garcia, S. , Sancho‐Balsells, A. , Longueville, S. , Herve, D. , Gruart, A. , Delgado‐Garcia, J. M. , Alberch, J. , & Giralt, A. (2020). Astrocytic BDNF and TrkB regulate severity and neuronal activity in mouse models of temporal lobe epilepsy. Cell death & Disease, 11(6), 411. 32483154 10.1038/s41419-020-2615-9 PMC7264221

[brb32594-bib-0018] Glien, M. , Brandt, C. , Potschka, H. , Voigt, H. , Ebert, U. , & Loscher, W. (2001). Repeated low‐dose treatment of rats with pilocarpine: Low mortality but high proportion of rats developing epilepsy. Epilepsy Research, 46(2), 111–119. 11463512 10.1016/s0920-1211(01)00272-8

[brb32594-bib-0019] Gordon, T. (2009). The role of neurotrophic factors in nerve regeneration. Neurosurgical Focus [Electronic Resource], 26(2), E3. 10.3171/FOC.2009.26.2.E3 19228105

[brb32594-bib-0020] Greenberg, M. E. , Xu, B. , Lu, B. , & Hempstead, B. L. (2009). New insights in the biology of BDNF synthesis and release: Implications in CNS function. Journal of Neuroscience, 9(41), 12764–12767. 10.1523/JNEUROSCI.3566-09.2009 PMC3091387 19828787

[brb32594-bib-0021] Gu, F. , Parada, I. , Shen, F. , Li, J. , Bacci, A. , Graber, K. , Taghavi, R. M. , Scalise, K. , Schwartzkroin, P. , Wenzel, J. , & Prience, D. A. (2017). Structural alterations in fast‐spiking GABAergic interneurons in a model of posttraumatic neocortical epileptogenesis. Neurobiology of Disease, 108, 100–114. 28823934 10.1016/j.nbd.2017.08.008 PMC5927780

[brb32594-bib-0022] Guo, W. , Robbins, M. T. , Wei, F. , Zou, S. , Dubner, R. , & Ren, K. (2006). Supraspinal brain‐derived neurotrophic factor signaling: A novel mechanism for descending pain facilitation. Journal of Neuroscience, 26(1), 126–137. 16399679 10.1523/JNEUROSCI.3686-05.2006 PMC6674294

[brb32594-bib-0023] Huang, E. J. , & Reichardt, L. F. (2003). Trk receptors: Roles in neuronal signal transduction. Annual Review of Biochemistry, 72, 609–642. 10.1146/annurev.biochem.72.121801.161629 12676795

[brb32594-bib-0024] Ito, K. , Kudo, M. , Sasaki, M. , Saito, A. , Yamashita, F. , Harada, T. , Yokosawa, S. , Uwano, I. , Kameda, H. , & Terayama, Y. (2016). Detection of changes in the periaqueductal gray matter of patients with episodic migraine using quantitative diffusion kurtosis imaging: Preliminary findings. Neuroradiology, 58(2), 115–120. 26446146 10.1007/s00234-015-1603-8

[brb32594-bib-0025] Iughetti, L. , Lucaccioni, L. , Fugetto, F. , Predieri, B. , Berardi, A. , & Ferrari, F. (2018). Brain‐derived neurotrophic factor and epilepsy: A systematic review. Neuropeptides, 72, 23–29. 30262417 10.1016/j.npep.2018.09.005

[brb32594-bib-0026] Jaseja, H. (2013). Pedunculopontine nucleus stimulation in intractable epilepsy: Simulation of nature's antiepileptic role and mechanism. Epilepsy & behavior : E&B, 27(3), 507. 10.1016/j.yebeh.2013.03.004 23578886

[brb32594-bib-0027] Jiang, D. G. , Jin, S. L. , Li, G. Y. , Li, Q. Q. , Li, Z. R. , Ma, H. X. , Zhuo, C. J. , Jiang, R. H. , & Ye, M. J. (2016). Serotonin regulates brain‐derived neurotrophic factor expression in select brain regions during acute psychological stress. Neural Regeneration Research, 11(9), 1471–1479. 27857753 10.4103/1673-5374.191222 PMC5090852

[brb32594-bib-0028] Junger, H. , & Sorkin, L. S. (2000). Nociceptive and inflammatory effects of subcutaneous TNFalpha. Pain, 85(1‐2), 145–151. 10692613 10.1016/s0304-3959(99)00262-6

[brb32594-bib-0029] Karatas, H. , Yemisci, M. , Eren‐Kocak, E. , & Dalkara, T. (2018). Brain Peptides for the Treatment of Neuropsychiatric Disorders. Current Pharmaceutical Design, 24(33), 3905–3917. 30417776 10.2174/1381612824666181112112309

[brb32594-bib-0030] Keay, K. A. , & Bandler, R. (1998). Vascular head pain selectively activates ventrolateral periaqueductal gray in the cat. Neuroscience Letters, 245(1), 58–60. 9596355 10.1016/s0304-3940(98)00168-2

[brb32594-bib-0031] Kim, D. W. , & Lee, S. K. (2017). Headache and Epilepsy. Journal of Epilepsy Research, 7(1), 7–15. 28775949 10.14581/jer.17002 PMC5540694

[brb32594-bib-0032] La Cesa, S. , Tinelli, E. , Toschi, N. , Di Stefano, G. , Collorone, S. , Aceti, A. , Francia, A. , Cruccu, G. , & Truini, A. (2014). Caramia F: FMRI pain activation in the periaqueductal gray in healthy volunteers during the cold pressor test. Magnetic Resonance Imaging, 32(3), 236–240. 24468081 10.1016/j.mri.2013.12.003

[brb32594-bib-0033] Lam, A. , Whelan, N. , & Corcoran, M. E. (2010). Susceptibility of brainstem to kindling and transfer to the forebrain. Epilepsia, 51(9), 1736–1744. 20384715 10.1111/j.1528-1167.2010.02551.x

[brb32594-bib-0034] Martins, L. B. , Teixeira, A. L. , & Domingues, R. B. (2017). Neurotrophins and migraine. Vitamins and Hormones, 104, 459–473. 28215304 10.1016/bs.vh.2016.10.003

[brb32594-bib-0035] McNamara, J. O. , & Scharfman, H. E. :(2012). Temporal lobe epilepsy and the BDNF receptor, TrkB. In: J. L., Noebels, M., Avoli, M. A., Rogawski, R. W., Olsen , & A. V., Delgado‐Escueta (Eds.), Jasper's basic mechanisms of the epilepsies. National Center for Biotechnology Information (US) . . 22787630

[brb32594-bib-0036] Menant, O. , Andersson, F. , Zelena, D. , & Chaillou, E. (2016). The benefits of magnetic resonance imaging methods to extend the knowledge of the anatomical organisation of the periaqueductal gray in mammals. Journal of Chemical Neuroanatomy, 77, 110–120. 27344962 10.1016/j.jchemneu.2016.06.003

[brb32594-bib-0037] Merighi, A. , Salio, C. , Ghirri, A. , Lossi, L. , Ferrini, F. , Betelli, C. , & Bardoni, R. (2008). BDNF as a pain modulator. Progress in Neurobiology, 85(3), 297–317. 18514997 10.1016/j.pneurobio.2008.04.004

[brb32594-bib-0038] Mitsikostas, D. D. , & Sanchez del Rio, M. (2001). Receptor systems mediating c‐fos expression within trigeminal nucleus caudalis in animal models of migraine. Brain Research Brain Research Reviews, 35(1), 20–35. 11245884 10.1016/s0165-0173(00)00048-5

[brb32594-bib-0039] Palma, E. , Torchia, G. , Limatola, C. , Trettel, F. , Arcella, A. , Cantore, G. , Di Gennaro, G. , Manfredi, M. , Esposito, V. , Quarato, P. P. , Miledi, R. , & Eusebi, F. (2005). BDNF modulates GABAA receptors microtransplanted from the human epileptic brain to Xenopus oocytes. PNAS, 102(5), 1667–1672. 15665077 10.1073/pnas.0409442102 PMC547850

[brb32594-bib-0040] Paradiso, B. , Marconi, P. , Zucchini, S. , Berto, E. , Binaschi, A. , Bozac, A. , Buzzi, A. , Mazzuferi, M. , Magri, E. , Navarro Mora, G. , Rodi, D. , Su, T. , Volpi, I. , Zanetti, L. , Marzola, A. , Manservigi, R. , Fabene, P. F. , & Simonato, M. (2009). Localized delivery of fibroblast growth factor‐2 and brain‐derived neurotrophic factor reduces spontaneous seizures in an epilepsy model. PNAS, 106(17), 7191–7196. 19366663 10.1073/pnas.0810710106 PMC2678472

[brb32594-bib-0041] Park, J. , Moon, H. , Akerman, S. , Holland, P. R. , Lasalandra, M. P. , Andreou, A. P. , Ferrari, M. D. , van den Maagdenberg, A. M. , & Goadsby, P. J. (2014). Differential trigeminovascular nociceptive responses in the thalamus in the familial hemiplegic migraine 1 knock‐in mouse: A Fos protein study. Neurobiology of Disease, 64, 1–7. 24355314 10.1016/j.nbd.2013.12.004

[brb32594-bib-0042] Porcaro, C. , Di Lorenzo, G. , Seri, S. , Pierelli, F. , Tecchio, F. , & Coppola, G. (2017). Impaired brainstem and thalamic high‐frequency oscillatory EEG activity in migraine between attacks. Cephalalgia, 37(10), 915–926. 27358281 10.1177/0333102416657146

[brb32594-bib-0043] Pradhan, A. A. , Smith, M. L. , McGuire, B. , Tarash, I. , Evans, C. J. , & Charles, A. (2014). Characterization of a novel model of chronic migraine. Pain, 155(2), 269–274. 24121068 10.1016/j.pain.2013.10.004 PMC3920577

[brb32594-bib-0044] Samineni, V. K. , Grajales‐Reyes, J. G. , Copits, B. A. , O'Brien, D. E. , Trigg, S. L. , Gomez, A. M. , Bruchas, M. R. , & Gereau, R. W. (2017). Divergent modulation of nociception by glutamatergic and GABAergic neuronal subpopulations in the periaqueductal gray. eNeuro, 4(2), . 10.1523/ENEURO.0129-16.2017 PMC5370278 28374016

[brb32594-bib-0045] Simonato, M. , Tongiorgi, E. , & Kokaia, M. (2006). Angels and demons: Neurotrophic factors and epilepsy. Trends in Pharmacological Sciences, 27(12), 631–638. 17055067 10.1016/j.tips.2006.10.002

[brb32594-bib-0046] Szucs, A. , Horvath, A. , Rasonyi, G. , Fabo, D. , Szabo, G. , Sakovics, A. , & Kamondi, A. (2015). Ictal analgesia in temporal lobe epilepsy—The mechanism of seizure‐related burns. Medical Hypotheses, 85(2), 173–177. 25953092 10.1016/j.mehy.2015.04.023

[brb32594-bib-0047] Tongiorgi, E. , Domenici, L. , & Simonato, M. (2006). What is the biological significance of BDNF mRNA targeting in the dendrites? Clues from epilepsy and cortical development. Molecular Neurobiology, 33(1), 17–32. 16388108 10.1385/MN:33:1:017

[brb32594-bib-0048] Vecchia, D. , & Pietrobon, D. (2012). Migraine: A disorder of brain excitatory‐inhibitory balance? Trends in Neurosciences, 35(8), 507–520. 22633369 10.1016/j.tins.2012.04.007

[brb32594-bib-0049] Wang, L. , Shen, J. , Cai, X. T. , Tao, W. W. , Wan, Y. D. , Li, D. L. , Tan, X. X. , & Wang, Y. (2019). Ventrolateral periaqueductal gray matter neurochemical lesion facilitates epileptogenesis and enhances pain sensitivity in epileptic rats. Neuroscience, 411, 105–118. 31158436 10.1016/j.neuroscience.2019.05.027

[brb32594-bib-0050] Wang, Y. , & Wang, X. S. (2013). Migraine‐like headache from an infarction in the periaqueductal gray area of the midbrain. Pain Medicine, 14(6), 948–949. 23565756 10.1111/pme.12096

[brb32594-bib-0051] Weidner, K. L. , Goodman, J. H. , Chadman, K. K. , & McCloskey, D. P. (2011). Aging‐induced seizure‐related changes to the hippocampal mossy fiber pathway in forebrain specific BDNF overexpressing mice. Aging and Disease, 2(4), 308–317. 22396883 PMC3295075

[brb32594-bib-0052] Yang, P. , Wang, Z. , Zhang, Z. , Liu, D. , Manolios, E. N. , Chen, C. , Yan, X. , Zuo, W. , & Chen, N. (2018). The extended application of the rat brain in stereotaxic coordinates in rats of various body weight. Journal of Neuroscience Methods, 307, 60–69. 29960030 10.1016/j.jneumeth.2018.06.026

[brb32594-bib-0053] Yang, P. S. , Peng, H. Y. , Lin, T. B. , Hsieh, M. C. , Lai, C. Y. , Lee, A. S. , Wang, H. H. , & Ho, Y. C. (2020). NMDA receptor partial agonist GLYX‐13 alleviates chronic stress‐induced depression‐like behavior through enhancement of AMPA receptor function in the periaqueductal gray. Neuropharmacology, 178, 108269. 32791085 10.1016/j.neuropharm.2020.108269

[brb32594-bib-0054] Yin, J. B. , Wu, H. H. , Dong, Y. L. , Zhang, T. , Wang, J. , Zhang, Y. , Wei, Y. Y. , Lu, Y. C. , Wu, S. X. , Wang, W. , & Li, Y. ‐ Q. (2014). Neurochemical properties of BDNF‐containing neurons projecting to rostral ventromedial medulla in the ventrolateral periaqueductal gray. Frontiers in Neural Circuits, 8, 137. 25477786 10.3389/fncir.2014.00137 PMC4238372

